# Patterning human neuronal networks on photolithographically engineered silicon dioxide substrates functionalized with glial analogues

**DOI:** 10.1002/jbm.a.34813

**Published:** 2013-06-11

**Authors:** Mark A Hughes, Paul M Brennan, Andrew S Bunting, Katherine Cameron, Alan F Murray, Mike J Shipston

**Affiliations:** 1 Centre for Integrative Physiology, School of Biomedical Sciences, The University of EdinburghEdinburgh, EH8 9XD, United Kingdom; 2 Edinburgh Cancer Research Centre, Institute of Genetics and Molecular Medicine, Western General HospitalEdinburgh, EH4 2XR, United Kingdom; 3 School of Engineering, Institute for Integrated Micro and Nano Systems, The University of EdinburghMidlothian, EH9 3JL, Edinburgh, United Kingdom

**Keywords:** parylene, photolithography, cell patterning, neuron, silicon dioxide

## Abstract

Interfacing neurons with silicon semiconductors is a challenge being tackled through various bioengineering approaches. Such constructs inform our understanding of neuronal coding and learning and ultimately guide us toward creating intelligent neuroprostheses. A fundamental prerequisite is to dictate the spatial organization of neuronal cells. We sought to pattern neurons using photolithographically defined arrays of polymer parylene-C, activated with fetal calf serum. We used a purified human neuronal cell line [Lund human mesencephalic (LUHMES)] to establish whether neurons remain viable when isolated on-chip or whether they require a supporting cell substrate. When cultured in isolation, LUHMES neurons failed to pattern and did not show any morphological signs of differentiation. We therefore sought a cell type with which to prepattern parylene regions, hypothesizing that this cellular template would enable secondary neuronal adhesion and network formation. From a range of cell lines tested, human embryonal kidney (HEK) 293 cells patterned with highest accuracy. LUHMES neurons adhered to pre-established HEK 293 cell clusters and this coculture environment promoted morphological differentiation of neurons. Neurites extended between islands of adherent cell somata, creating an orthogonally arranged neuronal network. HEK 293 cells appear to fulfill a role analogous to glia, dictating cell adhesion, and generating an environment conducive to neuronal survival. We next replaced HEK 293 cells with slower growing glioma-derived precursors. These primary human cells patterned accurately on parylene and provided a similarly effective scaffold for neuronal adhesion. These findings advance the use of this microfabrication-compatible platform for neuronal patterning. © 2013 The Authors. Journal ofBiomedicalMaterials Research Part APublished byWiley Periodicals, Inc.Wiley Periodicals, Inc. J Biomed Mater Res Part A: 102A: 1350–1360, 2014.

## INTRODUCTION

Engineering and interacting with bespoke *in vitro* neuronal networks have the potential to enhance understanding of information processing in real neuronal networks^1^ and may form a useful platform for pharmacological screening in diseases, such as epilepsy and stroke.^2^ As bidirectional interaction with such networks becomes possible, this also offers a promising approach to developing neuroprosthetic devices. However, creating such networks requires precise control of cell body adhesion and also neurite outgrowth and connectivity. To interact with a defined network, methods that enable stimulation and recording from patterned cells must also be amenable to incorporation. These collective demands motivate the approach of merging silicon semiconductor microelectronics with neuronal cell patterning.

The concept of building bespoke neuronal networks on silicon is not new.^3^–^5^ Contemporary work^6^ has furthered the idea to take advantage of various cellular lithographic techniques, has explored the impact of glia in patterned networks, and has utilized multielectrode arrays to record cellular activity. Our group focuses on the use of parylene-C as a neuronal patterning substrate. Parylene-C is a biocompatible polymer used commercially to coat printed circuit boards. Photolithographic patterning of parylene-C on silicon dioxide, followed by activation with serum, has enabled patterning of primary murine hippocampal cells,^7^–^10^ a human teratocarcinoma cell line,^11^,^12^ and the human embryonal kidney (HEK) 293 cell line.^13^ This straightforward and reliable technique is significantly simpler than some multistage protocols used for neuronal patterning. Specifically, patterned parylene substrates are biologically stable and can be stored until needed (whereupon they are activated).

Whilst parylene-C has been used previously in the context of cell patterning and cell trapping,^14^ its use for neuronal patterning after serum activation is in its infancy. Exploration of the mechanisms underlying cell patterning suggests that both adhesive and repulsive components in serum interact to imbue each substrate with contrasting cytoadhesive or cytorepulsive characteristics, although these components are not yet characterized.^13^

Although patterning primary murine hippocampal cells (which contain both neurons *and* glia) is effective, it remains unclear whether neurons in isolation are capable of patterning or whether glia adhere and (by close association) enable neurons to respect the underlying parylene geometry. The presence of glia amongst patterned neurons, though better reflecting the *in vivo* environment, may complicate downstream efforts to record from and stimulate individual neurons. We therefore sought to pattern neurons in isolation, questioning whether neurons themselves will pattern or whether they are dependent on the presence of glial (or other) cell types.

The lund human mesencephalic (LUHMES) cell line manifests well-described functional neuronal characteristics.^15^ These conditionally immortalized cells can be induced to differentiate by shutting down the *myc* transgene. Inactivation of the oncogene by tetracycline-mediated gene expression allows neuronal differentiation to proceed, resulting in a pure source of postmitotic neurons in 5 days. Important phenotypic characteristics include formation of one to two neurites (>500 µm long), dynamic growth cone behavior, and timely generation of spontaneous electrical activity.

We initially attempted to pattern isolated LUHMES in both their undifferentiated (UD) and their differentiated state. Subsequently, coculture environments were tested. These sought to assess neuronal behavior (with respect to cell adhesion and morphological differentiation) in the presence of a different prepatterned cell type. Toward this end, patterning behavior was assessed in a range of different cell lines and was quantified by measuring adhesive and repulsive indices on parylene and SiO_2_, respectively. Cell lines tested were

- UD N2a (Neuro 2A): a mouse neuroblastoma-derived neuronal cell line.
- HEK 293: previously considered a derivative of mouse embryonic fibroblastic or endothelial renal cells,^16^ current research suggests an early neuronal lineage (evidenced by presence of messenger RNA and gene products typically found in neurons neurofilament-M, neurofilament-L, and α-internexin) and the endogenous expression of several voltage-gated ion currents.^17^,^18^
- N9 microglia: a mouse-derived cell line with phenotypic characteristics similar to primary microglia.^19^
- UD 3T3 L1 preadipocytes: derived from 3T3 cells (themselves derived from primary mouse embryonic fibroblasts), these cells have fibroblast-like characteristics.^20^

HEK 293 cells illustrated the most robust patterning characteristics and were therefore used for initial coculture experiments with LUHMES neurons.

We subsequently assessed patterning characteristics of three different human glioma-derived stem-like cultures (GSC); our hypothesis being that these glial analogues would also pattern accurately on parylene. Finally, LUHMES neurons were sequentially cocultured with one of the human GSC lines. Our aim was to create a network of human neurons with a configuration defined by underlying human glial-like cells, with glial adhesion itself demarcated by parylene-patterned silicon dioxide.

## MATERIALS AND PROTOCOLS

### Fabrication of parylene patterns on SiO_2_: Process flow

1. Silicon wafers (Siltronix, Archamps, France) were oxidized in an atmospheric horizontal furnace (H_2_ 1.88 SLM and O_2_ 1.25 SLM) at 1100°C for 40 min to produce a 500 nm SiO_2_ layer (measured using a Nanometrics NanoSpec 3000 reflectometer).
2. Oxidized wafers were primed with Merck Silane A174 adhesion promoter, followed by deposition of 100 nm coating of parylene-C (22°C at a rate of 1.298 nm/mg of dimer using a SCS Labcoter 2 deposition Unit, Model PDS2010).
3. Hexamethyldisilazane adhesion promoter was deposited on parylene-coated wafers in a SVG 3 inch photoresist track followed by application of 1 µm thick film of Rohm & Hass SPR350-1.2 positive photoresist, by spinning at a speed of 4000 rpm for 30 s.
4. Wafers then soft baked for 60 s at 90°C.
5. Wafers and premanufactured photo mask (Compugraphics International, Glenrothes, Scotland) were placed in Suss Microtech MA/BA8 mask aligner and ultraviolet (UV) exposed. The primary parylene design consisted of three iterations of circular parylene nodes with a centered “cross-hair” (node diameters 250 µm, 100 µm, and 50 µm, cross hairs 450 µm in length for largest node size, 300 µm for the two smaller nodes) on chips 7.7 mm × 5.9 mm in dimension. All node/cross hair complexes were separated from one another by a distance of 100 µm. Nodes were arranged orthogonally with the chip designed such that there were three regions for each node size. A second chip design was used to further explore differences in on-chip behavior of GSC lines and LUHMES neurons. Here, 50 µm diameter nodes were arranged in a grid configuration separated from one another by 400 µm horizontally and vertically. Two different variations were created; one in which a 2-µm wide parylene track extended diagonally from node to node, and another in which only very short (30 µm long and 2 µm wide) parylene tracks partially extended from nodes.
6. Wafers were baked for 60 s at 110°C and the UV-exposed photoresist was removed by developing in Microchem MF-26A developer.
7. Wafers were inserted into a JLS RIE80 etch system for 120 s (50 mTorr chamber pressure, 49 sccm O_2_, 100 W RF power at 13.56 MHz) to etch off unprotected parylene (etch rate of 100 nm/min) and reveal underlying SiO_2_.
8. Wafers were cut with a DISCO DAD 680 Dicing Saw (spindle speed 30,000 rpm, feed speed 7 mm/s), rinsed in water, and blown dry with nitrogen.

### Chip cleaning and activation: Protocol

1. Residual photoresist was removed from all chips by brief immersion in acetone followed by rinsing in deionized distilled H_2_O three times.
2. Chips then immersed in piranha acid (5:3 ratio of 30% hydrogen peroxide and 98% sulfuric acid) for 10 min.
3. After a further deionized distilled H_2_O wash, chips were incubated for 3 h in fetal bovine serum (FBS; Gibco Invitrogen).

### Maintenance of cell lines: Protocol

1. HEK 293 cells (human embryonic kidney cells; American Type Culture Collection, VA) maintained at 37°C and 5% CO_2_ in Dulbecco's modified Eagle's medium (DMEM, Gibco Invitrogen) supplemented with 10% FBS. In monoculture experiments, cells applied to chips as a suspension of 5 × 10^4^ cells/mL in growth media and imaged live after 3 days *in vitro*.
2. LUHMES cells (American Type Culture Collection) maintained at 37°C and 5% CO_2_ in precoated plastic culture flasks (coated with 50 µg/mL poly-l-ornithine and 1 µg/mL fibronectin in H_2_O for 3 h). Proliferation media consisted of Advanced DMEM/F12 (Gibco Invitrogen), 1× N2 supplement, 2 m*M* l-glutamine and 40 ng/mL recombinant basic fibroblast growth factor (FGF; Gibco Invitrogen). Differentiation media consisted of Advanced DMEM/F12 (Gibco Invitrogen), 1× N2 supplement, 2 m*M* l-glutamine and 1 µg/mL tetracycline. To differentiate into postmitotic neurons, media changed from proliferation to differentiation 24 h after passage. After 2 further days in differentiation media, cells trypsinized and plated. For monoculture experiments, UD LUHMES plated as a suspension of 5 × 10^4^ cells/mL in proliferation media, predifferentiated LUHMES plated as a suspension of 30 × 10^4^ cells/mL in differentiation media and both imaged alive after 3 days *in vitro*.
3. 3T3 L1 (a gift from Dr Luke Chamberlain, Strathclyde Institute of Pharmacy and Biomedical Sciences, University of Strathclyde) maintained at 37°C and 10% CO_2_ in DMEM (Gibco Invitrogen) supplemented with 10% fetal calf serum and 1% Pen-Strep. Cells applied to chips as a suspension of 5 × 10^4^ cells/mL in growth media and imaged alive after 3 days *in vitro*.
4. N2a cells (American Type Culture Collection) maintained at 37°C and 5% CO_2_ in DMEM (Gibco Invitrogen) supplemented with 10% FBS. Plated on-chip at a density of 5 × 10^4^ cells/mL and imaged alive after 3 days *in vitro*.
5. N9 cells (a gift from Prof Alun Williams and Dr Clive Bate; The Royal Veterinary College, University of London) maintained at 37°C and 5% CO_2_ in Iscove's modified Dulbecco's medium (Gibco Invitrogen) with 5% FCS, 100 IU/mL penicillin, and 100 μg/mL streptomycin. Plated on-chip at a density of 15 × 10^4^ cells/mL and imaged alive after 3 days *in vitro*.
6. Human glioma-derived primary cell cultures were obtained from fresh human glioma tissue removed intraoperatively during surgery. All patients gave informed signed consent. The South East Scotland Research Ethics committee approved the study (LREC 2004/4/16). A single-cell suspension was generated from the tumor and cells were allowed to form neurospheres in nonadherent conditions, similar to previous descriptions of glioma stem cell (GSC) primary culture.^21^ GSCs are a subpopulation of cells within gliomas with stem-like characteristics and are thought to be responsible for tumor recurrence and treatment resistance. Briefly, cells were maintained at 37°C and 5% CO_2_ in Advanced DMEM F12 (1:1) (Gibco Invitrogen) supplemented with 1% B27 (10×), 0.5% N2 (100×), 1% Glutamax 100 m*M*, 1% penicillin–streptomycin, 1% fungizone, EGF 10 ng/mL (R&D Systems, Abingdon, UK), basic FGF 10 ng/mL (R&D Systems, Abingdon, UK), Heparin 5 µg/mL (Sigma Aldrich, Gillingham, UK). Once neurospheres had formed in suspension culture, cells were expanded on plastic flasks coated with growth factor reduced Matrigel (BD Biosciences, Oxford, UK) diluted 1:80 in Advanced DMEM F12 for 30 min at 37°C. In this way, GSC primary cultures were derived from three different tumors; one from a histologically-confirmed glioblastoma (named GSC-A) and two from oligodendroglioma (GSC-C and GSC-E). A 50-µL droplet containing 10,000 cells in suspension was pipetted onto the chip surface, incubated for 30 min followed by addition of maintenance media, and then imaged alive after 3 or 5 days *in vitro*.
7. Coculture protocols *HEK 293 with LUHMES*: HEK 293 cells were applied to chips as a suspension of 5 × 10^4^ cells/mL in growth media. 24 h later, media removed and chip transferred to fresh well. A 40-µL droplet containing 120,000 predifferentiated LUHMES was pipetted onto the chip surface, incubated for 30 min, followed by addition of LUHMES differentiation media. Cells were imaged daily up to 6 days *in vitro*. *GSC-A with LUHMES*: A 50 µL droplet containing 10,000 GSC cells in suspension was pipetted onto chip surface, incubated for 30 min, followed by addition of maintenance media. 72 h later, a 40-µL droplet containing 50,000 predifferentiated LUHMES was pipetted onto chip surface, incubated for 30 min, followed by addition of LUHMES differentiation media. Cells were imaged daily up to 6 days *in vitro*.
8. HEK 293 growth retardation/arrest protocols. *Growth retardation with citrinin*: Normal HEK 293 growth media was supplemented with citrinin (MP Biomedicals, Cambridge, UK) at a final concentration of 100 µ*M*.*Hypotonic cell stress*: dH_2_O was applied to HEK 293 cultures on parylene-patterned chips for time intervals of 30 s, 1 min, 3 min, 30 min, and 60 min before secondary LUHMES coculture.

### Cell imaging and analysis: Process

1. Live cell imaging *in vitro* using a Wild Heerbrugg (Switzerland) light microscope adapted for use with a Nikon Coolpix 4500 digital camera using an MDC2 relay lens. Image J (version 1.44o, National Institute for Health) was used for image analysis and measurement of cell surface areas.
2. Two indices were derived to assess contrasting aspects of cell patterning:
  *Parylene adhesion index* (PAI) was calculated by dividing the surface area of cell material on parylene by the total surface area of parylene within a given region of interest (ROI). Each ROI consisted of one iteration of the parylene geometric pattern (described earlier) surrounded by a square area of SiO_2_.
  *SiO_2_ repulsion index* (SRI) was calculated by dividing the surface area of cell material on SiO_2_ by the total area of SiO_2_ in a given ROI, and subtracting the result from 1. Hence, perfect patterning on parylene would result in a PAI of 1 (complete cell coverage of all parylene-patterned areas) and SRI of 1 (complete absence cell material from SiO_2_).
3. For each cell line, a minimum of 18 ROIs were interrogated (with equal representation of each of the three node diameters present on the chip and data pooled). Charted data is illustrated as means ± standard error of the mean (SEM). Mann–Whitney *U* tests were used to compare patterning indices across cell lines. Prism 5 for Mac OS X (GraphPad Prism Software, CA) was used for statistical analyses.
4. To quantify the impact of the parylene/SiO_2_ construct on neurite orientation, neurites in coculture experiments were traced manually using Image J. All neurites in a ROI were tracked from their start point (centered on a parylene node) to their end point (defined either as branching point, termination, or encountering another cell body). Traced segments were divided into 100 µm sub-segments. A tangent was taken to each sub-segment and the angle, θ, of each segment measured and categorized into 11.25° intervals. Entries 180° apart were summed, as the aim was to assess for neurite growth between the orthogonally arranged parylene nodes. A radial plot illustrating the frequency of θ values, according to their 11.25° categories, reveals the orientation of neurite segments. This process was conducted for areas encompassing each of the three different node geometries, and for differentiated LUHMES cultured randomly on a polystyrene surface treated to promote homogenous cell adhesion (with 50 µg/mL poly-l-ornithine and 1 µg/mL fibronectin in H_2_O for 3 h). 12 independent chip experiments enabled acquisition and measurement of 200 neurite subsegments (each of 100 µm length) representing each of the four different environments.Datasets derived for each of the four environments were compared using the Kolmogorov–Smirnov test.
5. For immunocytochemistry, cells were fixed with phosphate-buffered saline (PBS)/4% paraformaldehyde for 15 min at 20°C, then washed, permeabilized with PBS/0.2% Triton X-100 and preincubated with PBS/1% bovine serum albumin (Sigma Aldrich, MO) for 1 h at 20°C. After blocking, cocultured chips were incubated with Anti-Neuron-specific β-III Tubulin-NL637 conjugated antibody (R&D Systems, MN) at a final concentration of 1× (1:10 dilution) in blocking buffer for 3 h at room temperature. Chips patterned with glioma-derived primary cells in isolation were incubated with glial fibrillary acidic protein (1 µg/mL Alexa Fluor 488 anti-GFAP monoclonal antibody; Invitrogen). Chips were attached to glass slides and a cover slip mounted using Fluorsave reagent (Calbiochem, Merck Millipore, Germany). Chips were imaged with a Zeiss Axioskop microscope (Göttingen, Germany), using a ×10 oil objective with NA of 0.3. Image processing was carried out with Image J software.

## RESULTS

**Figure 1:**
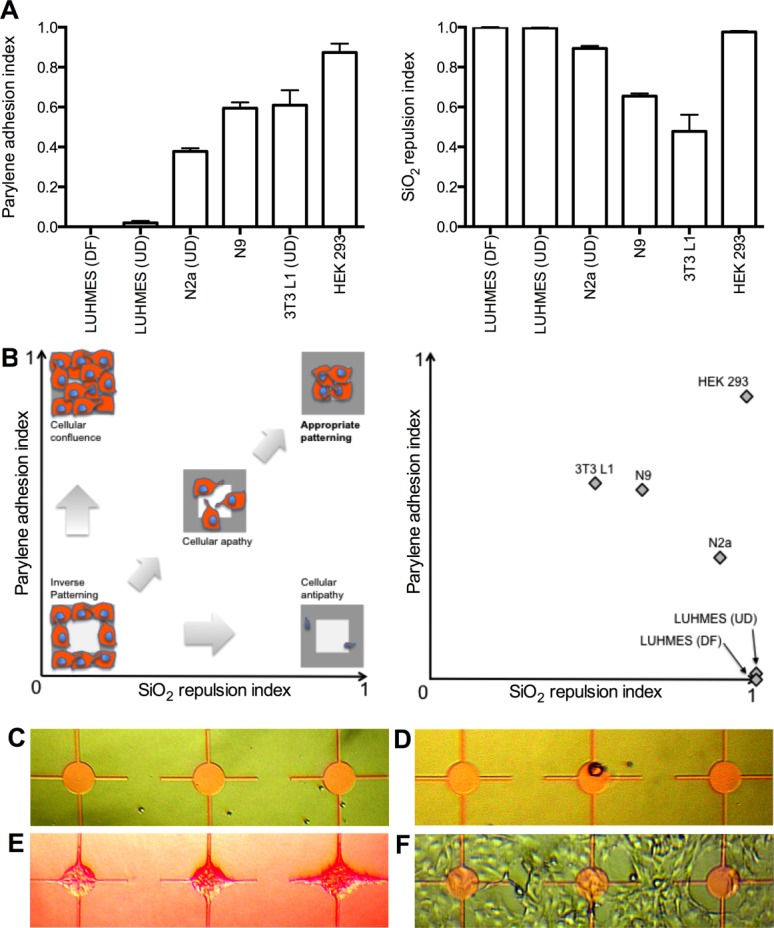
A, Histograms of parylene adhesion index and silicon repulsion index for predifferentiated LUHMES (DF), un-differentiated LUHMES (UD), undifferentiated N2a (UD), N9, undifferentiated 3T3 L1 (UD), and HEK 293 cell lines. Mean ± SEM. B, Cartoon illustrating range of cell patterning behaviors (left) and corresponding plot of cell lines tested (right). C, predifferentiated LUHMES. D, undifferentiated LUHMES. E, HEK 293. F, undifferentiated 3T3 L1. All images taken at 3DIV. Node diameter 100 µm. [Color figure can be viewed in the online issue, which is available at wileyonlinelibrary.com.]

UD and predifferentiated (DF) LUHMES failed to adhere, or show any morphological signs of differentiation, when cultured directly on-chip [UD LUHMES: PAI 0.02 ± 0.05, SRI 1.0 ± 0.01 (*n* = 27, assessed 3DIV); DF LUHMES: PAI 0.0 ± 0.0, SRI 1 ± 0.0 (*n* = 27, assessed 3DIV)]. This contrasts with robust adhesion and rapid differentiation of LUHMES when cultured on polystyrene precoated with poly-l-ornithine/fibronectin.^15^ Figure 1 illustrates LUHMES patterning behavior in comparison with other cell lines tested. The range of possible patterning behaviors can be demonstrated by plotting PAI against SRI. Figure 1(E) illustrates the high fidelity patterning behavior of the HEK 293 cell line, contrasting with the heterogeneous behavior of other cell lines tested.

**Figure 2:**
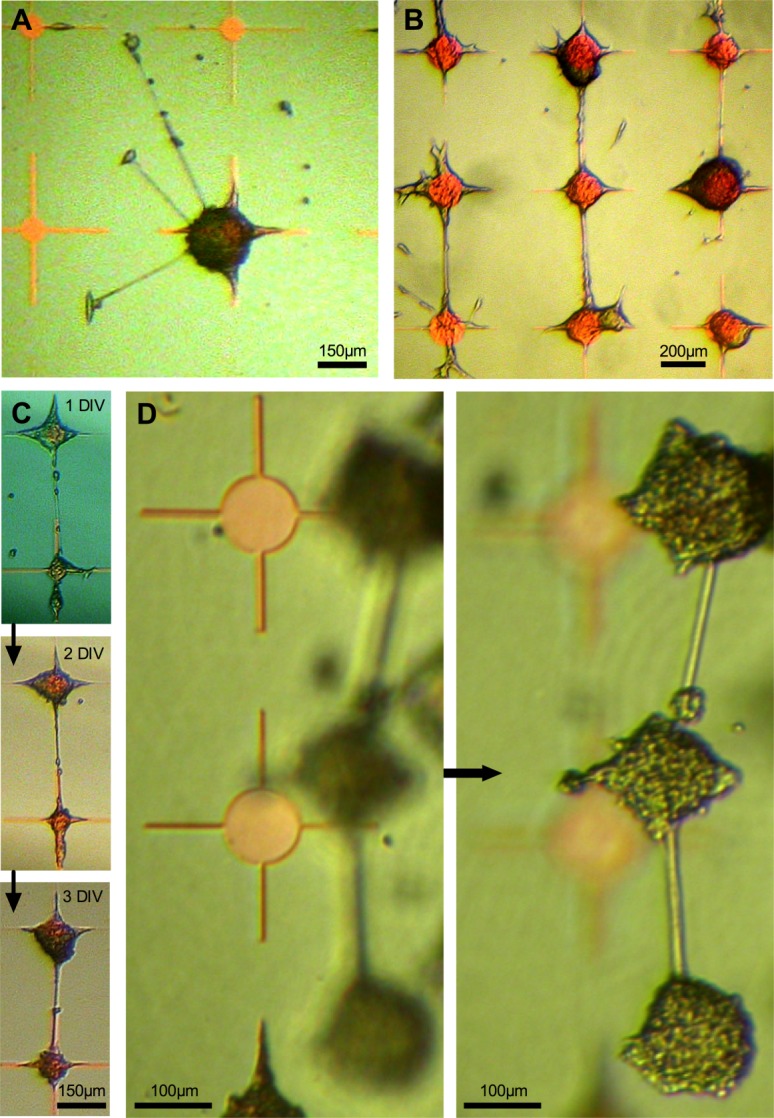
A and B, HEK 293 cells cocultured with predifferentiated LUHMES at 3DIV. C, Two interconnected nodes over 3 days, illustrating overgrowth of HEK 293 clusters. D, Neuronal network lift-off from chip surface (same region imaged at two different depths of focus) after 6DIV. [Color figure can be viewed in the online issue, which is available at wileyonlinelibrary.com.]

Although LUHMES fail to adhere in isolation, they are capable of patterning effectively if a chip is prepatterned with HEK 293 cells. Figure 2 illustrates LUHMES in coculture with HEK 293 cells. Prepatterned HEK 293 cells enable secondary adhesion of predifferentiated LUHMES. These neurons also show morphological signs of differentiation with neurites extending from parylene nodes to explore the surrounding SiO_2_ environment. The geometric arrangement of underlying parylene informs neuronal configuration, promoting formation of linear neurite connections between patterned nodes; as in Figure 2(B,C). Note, however, the continued rapid growth of underlying HEK 293 cells in Figure 2(C) from day one *in vitro* to day three. Figure 2(D) illustrates network lift-off after 6DIV.

When HEK 293 cells were grown on un-patterned polystyrene in media containing citrinin (to retard overgrowth), their doubling time was increased from 24.8 h to 63.4 h. However, in the context of patterned parylene substrates and LUHMES coculture, this reduction in proliferation was insufficient to prevent problematic node overgrowth.

Considering the possibility that patterned but nonviable HEK 293 cells (or cell membrane fragments) might still enable LUHMES adhesion, hypotonic cell stress was applied to HEK 293-patterned chips, before LUHMES application. HEK 293 cells lifted off extensively in response to all hypotonic stress time periods. Secondary application of LUHMES was globally unsuccessful with *no* adhesion to areas previously occupied by HEK 293 cells.

**Figure 3:**
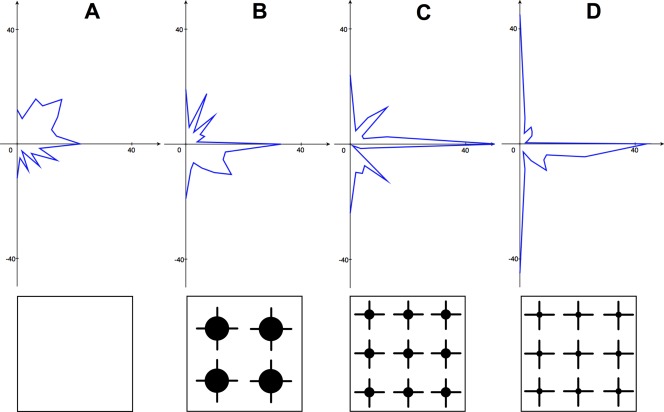
Directionality of neurite growth illustrated in radial plots. A, LUHMES on plain polystyrene, (B) coculture on 250 µm diameter parylene nodes, (C) coculture on 100 µm diameter nodes, and (D) coculture on 50 µm diameter nodes. [Color figure can be viewed in the online issue, which is available at wileyonlinelibrary.com.]

Directionality of neurite growth is shown in radial plots in Figure 3(A–D), illustrating each of the four different patterning environments (the three different node designs and unpatterned polystyrene surface treated to promote homogenous cell adhesion). Neurite directionality differs significantly according to environment (Kolmogorov–Smirnov tests: unpatterned vs. 250 µm diameter nodes *D* = 0.200, *P* = 0.001; 250 µm vs. 100 µm nodes *D* = 0.204, *P* = 0.001; 100 µm vs. 50 µm nodes *D* = 0.147, *P* = 0.028). There is a trend toward increasingly orthogonal growth as parylene configuration changes from 250 µm to 100 µm to 50 µm diameter nodes.

**Figure 4:**
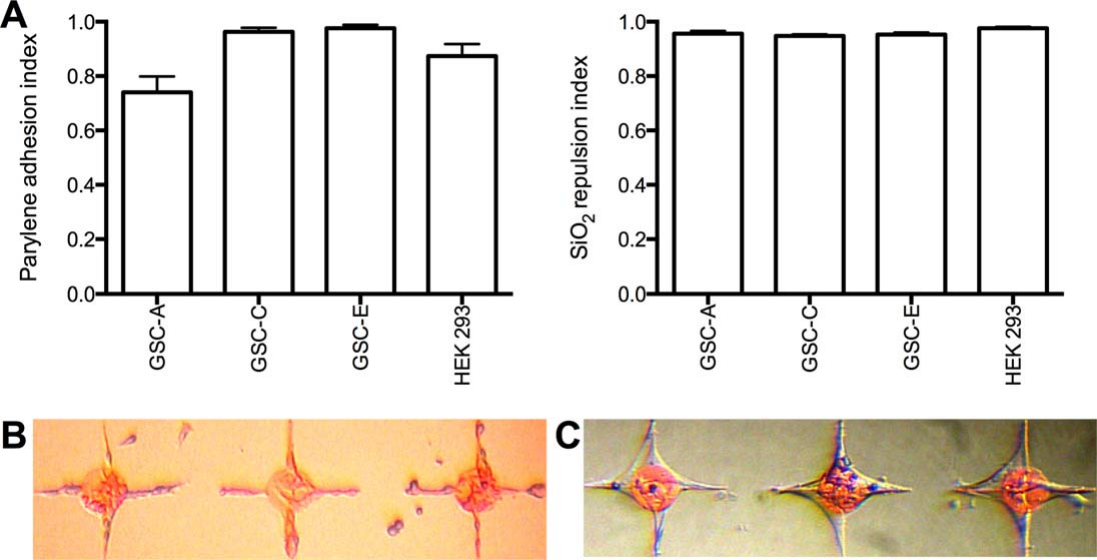
A, Histograms of parylene adhesion index and silicon repulsion index for tumor-derived glial cell lines: Glioblastoma line GSC-A, oligodendroglioma-derived lines GSC-C and GSC-E, and HEK 293 for comparison. B, Glioblastoma-derived GSC-A. C, Oligodendroglioma-derived GSC-C. All images taken at 3DIV. Node diameter 100 µm. [Color figure can be viewed in the online issue, which is available at wileyonlinelibrary.com.]

Figure 4(A–C) illustrates patterning behavior of glioma-derived glial cell lines. Both of the oligodendroglioma-derived lines GSC-C and GSC-E [line C: PAI 0.96 ± 0.01, SRI 0.95 ± 0.01 (*n* = 18, assessed 3DIV) and line E: PAI 0.98 ± 0.01, SRI 0.95 ± 0.01 (*n* = 18, assessed 3DIV)] and the glioblastoma-derived GSC-A [PAI 0.74 ± 0.06, SRI 0.96 ± 0.01 (*n* = 18, assessed 5DIV)] show excellent patterning characteristics; comparable to those exhibited by HEK 293 cells.

**Figure 5:**
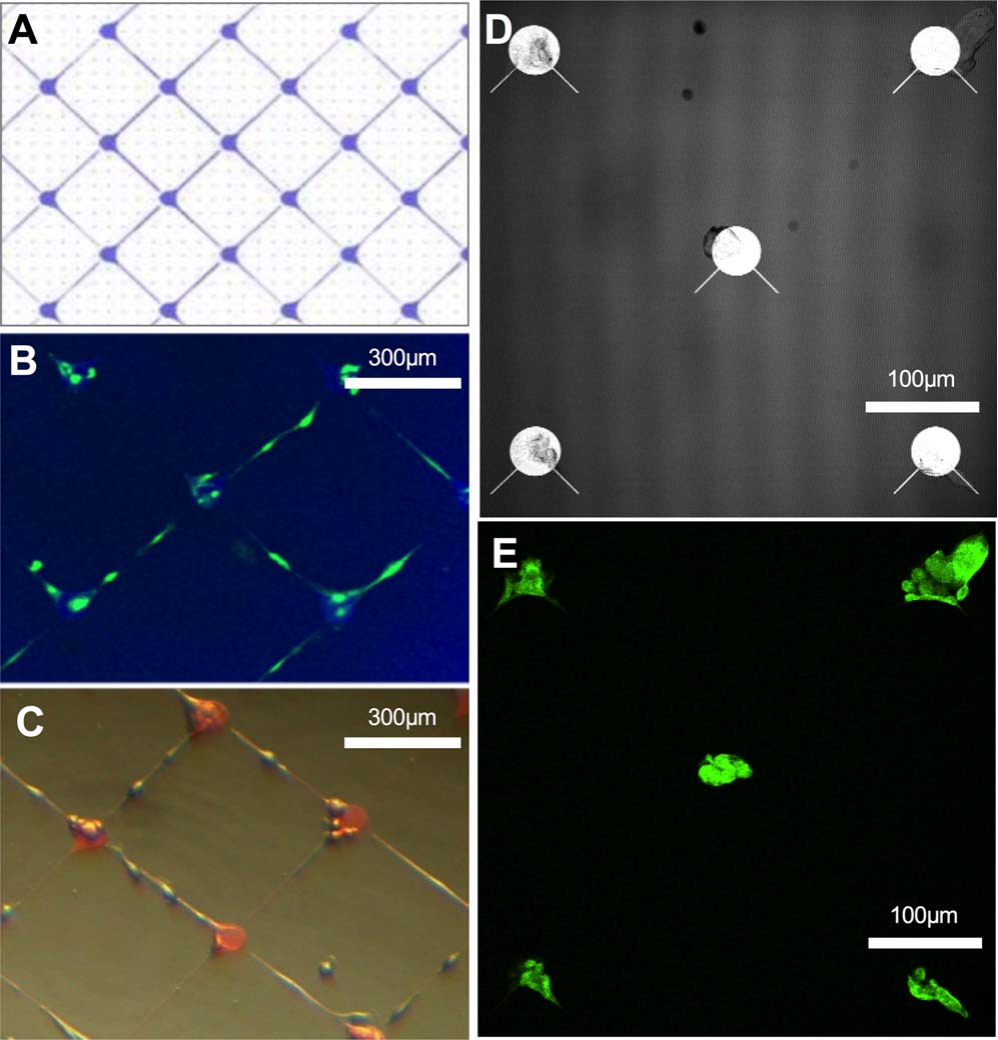
GSC-A cell line cultured on-chip in isolation. A, Schematic of parylene pattern imaged in (B) and (C). B, GSC-A fixed and stained with glial fibrillary acidic protein. C, GSC-A imaged alive at 3DIV. D, Reflectance image showing parylene pattern in (E). E, GSC-A cells fixed and stained with glial fibrillary acidic protein. [Color figure can be viewed in the online issue, which is available at wileyonlinelibrary.com.]

**Figure 6:**
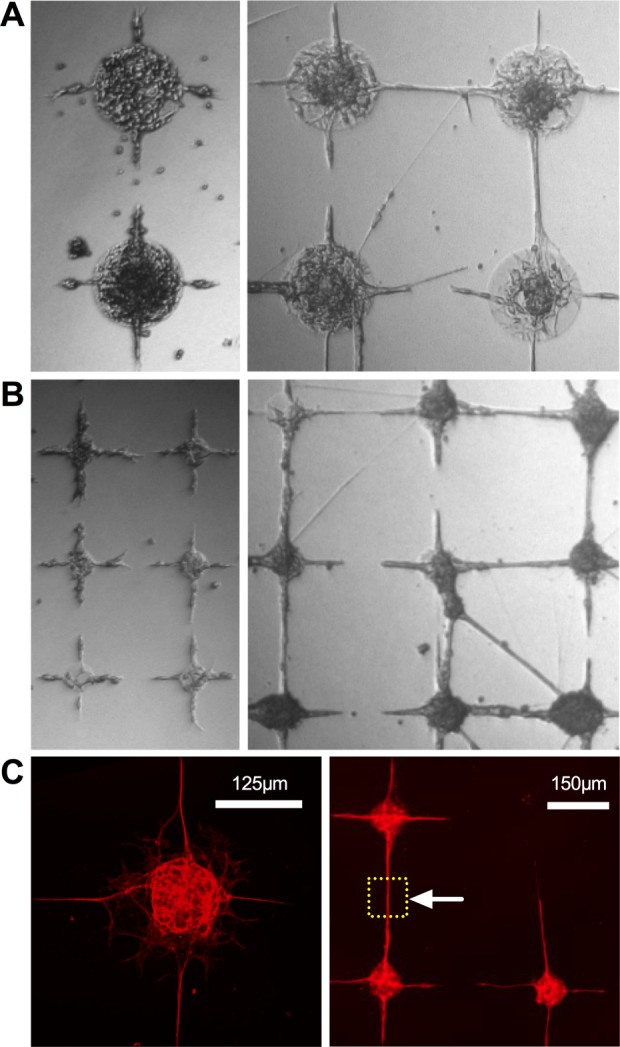
A and B, Glioblastoma-derived line GSC-A in isolation (left) and in coculture with predifferentiated LUHMES (right). Node diameter 250 µm in (A) and 100 µm in (B). C, Neuron-specific β-3 tubulin stained coculture of predifferentiated LUHMES with glioblastoma-derived glial cell GSC-A. Fixed after 5DIV. The dashed square marked by the arrowhead demarcates a neurite-only region of interest. [Color figure can be viewed in the online issue, which is available at wileyonlinelibrary.com.]

Figures 5(A–E) and 6(A,B, left panels) illustrate GSC-A cells in isolation on-chip. GSC-A cells pattern reliably on parylene but are not able to bridge the gap between parylene nodes. Only when the chip pattern is altered to provide a parylene track will GSC-A cells be induced to connect between nodes [compare Fig. 5(A–C) with (D,E)]. In contrast, when LUHMES are cocultured on GSC-A, they illustrate the capacity to extend neurites between nodes [compare left and right panels of Fig. 6(A,B)]. Figure 6(C) shows neuron-specific βIII tubulin-stained cocultures, confirming the presence of neurites projecting between nodes.

## DISCUSSION

### LUHMES in isolation

We set out to establish whether isolated LUHMES neurons were capable of selective adhesion and network formation on activated parylene-patterned SiO_2_ substrates. Isolated LUHMES do not pattern, either in an UD or differentiated state, nor do they manifest morphological changes suggesting differentiation (Fig. 1). For LUHMES the chip constitutes a globally cell repulsive environment, reflected by the very low PAI and very high SRI. This behavior contrasts starkly with the accurate patterning seen with primary murine hippocampal cells,^7^–^9^ suggesting that the presence of glia in these preparations may be key to enabling neuronal patterning.

### Heterogeneous patterning behavior across cell lines

We next explored patterning behavior across a range of cell lines, confirming the heterogeneity of responses toward the parylene/SiO_2_ chip construct (Fig. 1). The interface between a cell and adjacent foreign substrate is complex, dynamic, and bidirectional.^22^ Having illustrated this variation in cell adhesion behavior, a future strategy may involve identifying differences in the expression of key cell surface adhesion molecules that contribute to a cell's capacity to pattern. For example, interrogation of DNA microarray databases from a variety of cell types with contrasting patterning behavior should allow correlation between gene expression profiles and a cell's tendency to adhere to or be repulsed by the two contrasting substrates. This may open the way to targeted cell manipulation so as to *empower* a cell to pattern. Moreover, this approach may reciprocally help to elucidate important substrate-specific aspects of patterning.

### Coculture of LUHMES and HEK 293 cells

Given the failure of isolated LUHMES to pattern we questioned whether neurons would pattern in the context of a supporting cell substrate, reminiscent of the glia-neuron patterning seen with primary rodent cocultures.^7^–^9^ Of the array of four cell lines tested, HEK 293 cells manifest the best patterning profile and were used for initial coculture experiments. HEK 293 cells enable LUHMES to adhere selectively to the chip, by binding with patterned HEK cell clusters on parylene. As such, HEK 293 cells appear to perform a role analogous to glia. Moreover, LUHMES neurons that attach to HEK 293 cell clusters differentiate morphologically, evidenced by neurite formation. HEK 293 cells facilitate neuronal patterning by providing a physical point of attachment on-chip. In addition, this cell:cell interaction may enable LUHMES differentiation to proceed, where this was not possible in isolation.

### Control of neurite behavior

For the creation of neuronal networks capable of meaningful interrogation, neurite growth direction and connectivity needs to be controlled. We illustrated the ability to grow orthogonally arranged networks, as defined by the geometric configuration of parylene nodes. Each of the three node sizes/configurations used promotes some orthogonality but accuracy improves from largest (250 µm diameter) to smallest (50 µm diameter) node.

Neurite growth cones, in contrast with LUHMES and HEK 293 cell somata, appear capable of adhering and traversing unpatterned SiO_2_. It is unclear whether on-chip neurite growth is dominated by haptotactic or chemotactic (or both) mechanisms. Neurites appear to explore surrounding areas [Fig. 2(A)] and, after encountering another node, straighten up under tension. That growth cones of developing neurons generate tensile forces is well described^23^ and this self-organizing behavior has been observed before in the context of carbon–nanotube patterned substrates.^1^ Recent work using primary hippocampal cells has also noted how the geometry of patterned adhesive regions can influence axonal outgrowth,^24^ in this case using different polygons of microcontacted printed poly-l-lysine and laminin.

Although this approach allows a degree of network organization, it is hindered by the rapid proliferation of HEK 293 cells. As unrestricted HEK 293 proliferation continues, in contrast with arrested growth of postmitotic LUHMES neurons, patterning is overwhelmed. The combination of HEK 293 overgrowth, and the internode tension applied by linking neurites, tends to cause cell lift off and network obliteration over time (6–7DIV). This is an insufficient time window for the establishment of spontaneous electrical activity of neurons in the network. In addition, our ability to reliably fix and stain LUHMES/HEK 293 cocultures was compromised due to network fragility and cell lift-off. To ameliorate, methods for retarding or arresting HEK cell growth were explored. Citrinin (a nephrotoxic mycotoxin which disrupts microtubule function) has been used to induce cell cycle arrest in HEK 293 cells.^25^ Citrinin successfully reduced HEK 293 doubling time on unpatterned polystyrene substrates but its effect was insufficient in the context of patterned coculture. However, such network lift-off [seen best in Fig. 3(D)] is potentially useful for neuroregeneration purposes. For example, it opens the possibility of creating a neuronal network on-chip that could then be detached and implanted with the intention of *in vivo* nervous system repair.

Questioning the mechanics by which HEK 293 cells facilitate LUHMES adhesion, we used hypotonic cell stress before LUHMES application. Hypotonic cell stress using dH_2_O for intervals ranging from 30 s to 60 min resulted in almost total HEK 293 cell lift off. However, secondary application of LUHMES was universally unsuccessful, suggesting residual cell fragments/membranes are insufficient to enable LUHMES adhesion.

### Coculture of LUHMES and glial analogues

Given the success of HEK 293 cells as a glial analogue, a source of human glial cells was sought to improve the patterning model and potentially circumvent the issue of cell overgrowth. Our group has previously established primary cultures of human glial stem-like cells from a range of human gliomas. Cells from these cultures have been shown to have predominantly astrocytic characteristics, but also have tumor-associated mutations. Of the three primary cultures GSC-C and GSC-E have a much less disordered genome than GSC-A, because GSC-A is derived from a higher grade tumor. Despite their genetic aberrations, and evident ineligibility for downstream uses including neuroprosthetics, these cultures are more readily available than primary human astrocytes and easier to work with at this proof-of-concept stage. They also grow more slowly than the serially passaged HEK 293 cells, facilitating better observation and intervention of the glial–neuronal interaction.

Given previously published primary hippocampal cell work, we first hypothesized that these glial cell lines *would* pattern effectively. This is confirmed (in Fig. 4) with excellent patterning indices, comparable to those of HEK 293 cells. Using GSC-A to prepattern the parylene template, we again illustrated successful coculture with LUHMES and the generation of reticular orthogonally arranged networks (see Fig. 4). GSC-A cells are slower growing than HEK 293 cells, providing a greater opportunity for long-term establishment of stable neuronal networks. Glioma-derived cell lines and the HEK 293 cell line robustly respect the underlying parylene design; being repulsed from bare SiO_2_ and adhering to parylene. Only by creating a design with a parylene track between nodes can GSC-A be induced to connect with adjacent nodes. This contrasts with neurite behavior; with neurites being capable of bridging the nonparylene gap. This is important for downstream techniques to interrogate electrical and synaptic activity in the patterned network. By focusing on the parylene-free internode gap [Fig. 6(C), region demarcated by arrow head] one can be confident that neurites alone are being interrogated.

Future work will seek to better establish the rules governing neurite growth and organization. Axon guidance molecules or other topographical features on-chip may allow another level of control. Work is ongoing to assess electrophyiological and other functional behaviors of patterned neurons, and to confirm formation of functional synapses in the network. In addition, improved understanding of the underlying mechanisms governing cell adhesion to activated parylene will further enhance the utility of this platform.

## CONCLUSIONS

LUHMES neurons require an intermediate cell type to adhere to serum-activated parylene-patterned SiO_2_. HEK 293 cells fulfill this role and, in doing so, perform a function analogous to glia. These engineered cocultures organize into orthogonally configured reticular networks, informed by the spatial geometry of underlying parylene nodes. However, HEK 293 overgrowth, neurite tensile forces, and resultant cell lift-off compromise long-term *in vitro* network viability.

Human tumor-derived glial precursor cells pattern accurately and directly on serum-activated parylene-patterned SiO_2_. These prepatterned glial analogues enable defined secondary adhesion of LUHMES neurons, and in so doing promote formation of orthogonally arranged neurite connections. This work illustrates the potential utility of activated parylene as another tool for generation of bespoke neuronal networks on silicon.

## References

[b1] Shein-Idelson M, Ben-Jacob E, Hanein Y (2011). Engineered neuronal circuits: A new platform for studying the role of modular topology. Front Neuroeng.

[b2] Biffi E, Piraino F, Pedrocchi A, Fiore GB, Ferrigno G, Redaelli A, Menegon A, Responi M (2012). A microfluidic platform for controlled biochemical stimulation of twin neuronal networks. Biomicrofluidics.

[b3] Thomas CA, Springer PA, Okun LM, Berwald Y, Loeb GE (1972). A miniature microelectrode array to monitor the bioelectric activity of cultured cells. Exp Cell Res.

[b4] Pine J (1980). Recording action potentials from cultured neurons with extracellular micro-circuit electrodes. J Neurosci Methods.

[b5] Gross GW (1979). Simultaneous single unit recording in vitro with a photoetched laser deinsulated gold multimicroelectrode surface. IEEE Trans Biomed Eng.

[b6] Wheeler BC, Brewer GJ (2010). Designing neural networks in culture. Proc IEEE Inst Electr Electron Eng.

[b7] Delivopoulos E, Murray AF, MacLeod NK, Curtis JC (2009). Guided growth of neurons and glia using microfabricated patterns of parylene-C on a SiO_2_ background. Biomaterials.

[b8] Delivopoulos E, Murray AF, Curtis JC (2010). Effects of parylene-C photooxidation on serum-assisted glial and neuronal patterning. J Biomed Mater Res A.

[b9] Delivopoulos E, Murray AF (2011). Controlled adhesion and growth of long term glial and neuronal cultures on parylene-C. PLoS One.

[b10] Unsworth CP, Delivopoulos E, Gillespie T, Murray AF (2011). Isolating single primary rat hippocampal neurons & astrocytes on ultra-thin patterned parylene-C/silicon dioxide substrates. Biomaterials.

[b11] Unsworth CP, Holloway H, Delivopoulos E, Murray AF, Simpson MC, Dickinson ME, Graham ES (2011). Patterning and detailed study of human hNT astrocytes on parylene-C/silicon dioxide substrates to the single cell level. Biomaterials.

[b12] Unsworth CP, Graham ES, Delivopoulos E, Dragunow M, Murray AF (2012). First human hNT neurons patterned on parylene-C/silicon dioxide substrates: Combining an accessible cell line and robust patterning technology for the study of the pathological adult human brain. J Neurosci Methods.

[b13] Hughes MA, Bunting A, Cameron K, Murray AF, Shipston MJ (2013). Modulating patterned adhesion and repulsion of HEK 293 cells on micro-engineered parylene-C/SiO_2_ substrates. J Biomed Mater Res A.

[b14] Tan CP, Craighead HG (2010). Surface engineering and patterning using parylene for biological applications. Materials.

[b15] Scholz D, Pöltl D, Genewsky A, Weng M (2011). Rapid, complete and large-scale generation of post-mitotic neurons from the human LUHMES cell line. J Neurochem.

[b16] Graham FL, Smiley J, Russell WC, Nairn R (1977). Characteristics of a human cell line transformed by DNA from human adenovirus type 5. J Gen Virol.

[b17] Shaw G, Morse S, Ararat M, Graham FL (2002). Preferential transformation of human neuronal cells by human adenoviruses and the origin of HEK 293 cells. FASEB J.

[b18] Zhu G, Zhang Y, Xu H, Jiang C (1988). Identification of endogenous outward currents in the human embryonic kidney (HEK 293) cell line. J Neurosci Methods.

[b19] Righi M, Mori L, De Libero G, Sironi M, Biondi A, Mantovani A, Donini SD, Ricciardi-Castagnoli P (1989). Monokine production by microglial cell clones. Eur J Immunol.

[b20] Green H, Meuth M (1974). An established pre-adipose cell line and its differentiation in culture. Cell.

[b21] Pollard SM, Yoshikawa K, Clarke ID, Danovi D, Stricker S, Russel R, Bayani J, Head R, Lee M, Bernstein M, Squire JA, Smith A (2009). Glioma stem cell lines expanded in adherent culture have tumor-specific phenotypes and are suitable for chemical and genetic screens. Cell Stem Cell.

[b22] Streuli CH, Akhtar N (2009). Signal co-operation between integrins and other receptor systems. Biochem J.

[b23] Lowery LA, Van Vactor D (2009). The trip of the tip: Understanding the growth cone machinery. Nat Rev Mol Cell Biol.

[b24] Yang JM, Nam Y (2012). Geometric effect of cell adhesive polygonal micropatterns on neuritogenesis and axon guidance. J Neural Eng.

[b25] Chang CH, Yu FY, Wu TS, Wang LT, Liu BH (2011). Mycotoxin citrinin induced cell cycle G2/M arrest and numerical chromosomal aberration associated with disruption of microtubule formation in human cells. Toxicol Sci.

